# Preparation of Ultra-High Temperature Resistant Cyclodextrin-Based Filtration Loss Reducer for Water-Based Drilling Fluids

**DOI:** 10.3390/molecules29122933

**Published:** 2024-06-20

**Authors:** Yilin Liu, Lesly Dasilva Wandji Djouonkep, Boyang Yu, Chenyang Li, Chao Ma

**Affiliations:** 1 College of Petroleum Engineering, Yangtze University, Wuhan 430100, China; 15079171252@aliyun.com (Y.L.);; 2 Key Laboratory of Drilling and Production Engineering for Oil and Gas, Yangtze University, Wuhan 430100, China

**Keywords:** fluid loss reducer, drilling fluid, high temperature resistance, cyclodextrin

## Abstract

In the development of ultra-deep wells, extremely high temperatures can lead to inefficiency of additives in drilling fluids. Hence, there is a need to prepare additives with a simple preparation process and good effects at ultra-high temperatures to ensure stable drilling fluid performance. In this study, a high temperature resistant filtration loss polymer (LY-2) was prepared using γ-methacryloyloxypropyltrimethoxysilane (KH570), N,N-dimethylallyl ammonium chloride (DMDAAC), sodium p-styrenesulfonate (SSS), and β-cyclodextrin (β-CD). The impact of the different monomer ratios on particle size, rheology, and filtration performance was systematically investigated. Infrared spectroscopy afforded the structural features. Thermogravimetric Analysis detected the temperature stability, and scanning electron microscopy characterized the polymer micromorphology. LY-2 was completely decomposed at a temperature above 600 °C. Experiments showed FL_API_ of the drilling fluid containing 3% LY-2 aged at 260 °C/16 h was only 5.1 mL, which is 85.4% lower compared to the base fluid. This is attributed to the synergistic effect of the polymer adsorption through chemical action at high temperatures and the blocking effect of carbon nanoparticles on the filter cake released by cyclodextrin carbonization at high temperatures. Comparing LY-2 with commercial filter loss reducers shows that LY-2 has excellent temperature resistance, which exhibited five times higher filtration performance and relatively low cost, making it possible to be applied to ultra-high temperature drilling operations in an industrial scale-up.

## 1. Introduction

With the rising consumption of oil and gas resources in recent years, reserves of shallow strata are depleting rapidly. Consequently, petroleum exploration and development have shifted focus from shallow to deep strata. This change in approach has led to an increase in well depth, resulting in more frequent encounters with deep and ultra-deep wells. These encounters often involve challenging formations and higher temperatures at the well bottom. Hence, drilling fluids must perform optimally even in complex and high temperature conditions [1,2,3,4,5,6].

Drilling fluids have three types: water-based, oil-based, and synthetic-based drilling fluids. Oil-based drilling fluids offer excellent wellbore stability but are limited in use due to high costs and strict environmental regulations. Synthetic-based drilling fluids, also known as low-toxicity oil-based mud, use synthetic fluid instead of petroleum as the external phase of the reversed-phase emulsified mud, which is more environmentally friendly compared to oil-based drilling fluids, and the cost is also higher. Water-based drilling fluids consist of water and various additives such as filter loss reducers, encapsulants, viscosity reducers, lubricants, inhibitors, and weighting materials [7,8,9]. Among these additives, the filter loss reducer is particularly important as it helps maintain the drilling fluid’s stable rheological properties and helps with the formation of a smooth and dense mud cake, and it prevents excessive penetration of drilling fluids into formations, which not only maintain the stability of the wellbore but also helps facilitate the suspension and release of rock cuttings [10,11,12,13]. However, water-based drilling fluids may present challenges when encountering water-sensitive formations during drilling. For instance, uncontrolled filtrate may penetrate deeper into the formation, and as a consequence, it might dissolve formation minerals and cause wellbore instability and formation damage [14]. Additionally, changes in the mud’s rheological properties can negatively impact the drilling efficiency. So, it is important to keep the drilling fluid’s rheological properties stable and minimize filtration loss.

Filtration loss reducers are widely used in drilling fluids, mainly consisting of natural polymers and synthetic polymers [15,16]. Natural polymers are usually effective in maintaining the performance of drilling fluids at low temperatures; they include xanthan gum, guar gum, carboxymethyl cellulose, lignin, chitosan, gelatin, natural rubber, humic acid, starch, and so on. Natural materials appear as a safe, more cost-effective, and environmentally friendly option. Nowadays, many scholars have attempted to modify natural polymers to further enhance their properties. Wang et al. [17] found that, through sulfonation modification, graft modification, compounding or complexation modification, and crosslinking modification, they can improve the temperature and salt resistance, improve the rheology, and make the drilling fluid performance more stable. According to Song et al. [18], incorporating nanocellulose into drilling fluids increases viscosity, improves non-Newtonian fluid properties, and effectively controls filtration loss. Following this logic, Zoveidavianpoor et al. [19] developed nanoscale tapioca starch as a water-soluble polymer for water-based drilling fluids and showed that the NPs acted as an efficient filtration control agent with improved performance in terms of viscosity, yield point, gelling strength, and rheological control, but the application of the natural material is limited by thermal stability, and the modification of natural polymers to improve temperature resistance has become a popular research topic. Wang et al. [20,21,22] successfully obtained hydrophobically-conjugated hydroxyethyl cellulose by grafting it onto the surface of calcium carbonate nanoparticles. The resulting copolymer showed excellent properties in reducing filtration loss up to 180 °C.

Starch is a widely used mud additive due to its polyhydroxyl functional groups. Jiang et al. [23,24] prepared modified carboxymethyl starch (CBF) through graft copolymerization, demonstrating good stability in brine mud at a high temperature of 150 °C. Wang et al. [25] introduced sodium silicate to create a novel inorganic silica-modified carboxymethyl starch filter loss reducer (Si-SCMS), which resulted in a filtration loss of only 15.2 mL after hot rolling at 150 °C. Dias et al. [26] investigated the effect of modified starch composition and its performance as a filter loss agent in inverse emulsion drilling fluids at 160 °C. Sagitov et al. [27] evaluated the filtration loss reduction effect of carboxymethylated starch and found that its performance mimics those of low-viscosity polyanionic cellulose (PAC) but at a 30% to 50% lower cost. Hence, carboxymethylated starch can be used as a substitute for PAC in the drilling industry. Ricky et al. [28] prepared a modified corn starch (MCS) that significantly improved the rheological and loss control properties of water-based drilling fluids at 0.3 wt% after aging at 220 °C. Cyclodextrin is a kind of starch-derivative; and after 1975, as the cost of industrial production decreased, research on the application of cyclodextrins continued to increase. There are three main types of cyclodextrins in nature according to the size of the ring diameter in this order: α-cyclodextrin, β-cyclodextrin, and γ-cyclodextrin. In industrial production, cyclodextrins can be obtained by acid hydrolysis, enzymatic hydrolysis, and chemical synthesis using three methods [29,30,31]. The method of synthesizing cyclodextrins through the chemical route is more costly and is used more for laboratory research. Enzymatic hydrolysis production, on the other hand, not only can produce high-purity cyclodextrins efficiently but also has less impact on the environment, as many microorganisms can use it as a carbon source and decompose it into small molecules such as glucose and carbon dioxide. Compared with some synthetic polymers that are difficult to degrade, cyclodextrins can be broken down relatively quickly in the environment, thus reducing environmental pollution. It is a non-toxic industrial product. With the in-depth research on cyclodextrins, their industrial applications, such as in the pharmaceutical field, food industry, textile industry, environmental protection field, catalytic field, materials science, etc., are expanding, and cyclodextrin-based metal-organic framework materials are also playing an important role in the fields of food packaging, drug delivery, sensors, adsorbents, and membrane materials [29]. All these applications reflect the multifunctionality of cyclodextrins [32,33,34,35]. In the early 1980s, cyclodextrin-based materials began to be used in oilfields. The difference between β-cyclodextrin and starch is that cyclodextrin is formed by six glucose molecules connected at the head and tail and has a special cone-shaped ring-table shape structure, which gives it good temperature resistance properties. Therefore, because of its stable chair conformation, [31,33,36,37] barrel structure with cavities, as well as its environmental friendliness, cost-effectiveness, and affordability, β-Cyclodextrin has attracted much attention from scholars. Zhong et al. [38] discovered that by preparing cyclodextrin polymer microspheres (β-CDP), β-CDP exhibits a “temperature-responsive” characteristic, which demonstrated good loss reduction performance above 160 °C. The results indicated that cyclodextrin can be used as a filtration control additive for higher-temperature applications.

Using organic monomers to synthesize filtration reducers was a significant advancement to the temperature resistance of polymers. Commonly used monomers in synthetic polymers include polyacrylamide, polyvinyl alcohol, polyacrylic acid, ethylene oxide polymer, etc. [39,40,41,42]. Furthermore, compounds such as 2-acrylamido-2-methylpropanesulfonic acid (AMPS) and acrylamide (AM) can increase the hydration layer repulsion by adsorption of bentonite particles, leading to the dispersion of bentonite particles and, consequently, maintain better stability of the drilling fluid. These polymers have good properties for modifying rheology and controlling filtration at temperatures below 200 °C. However, their properties are compromised by high salt concentrations and temperatures above 200 °C. Similarly, sodium p-styrenesulfonate (SSS) monomer has attracted attention in the petroleum industry due to its excellent thermal stability and strong hydration properties. Wang et al. [43] developed a filtrate reducing agent called DANS, which is resistant to ultra-high temperatures and salt contamination. They used N, N-dimethylacrylamide (DEAM), AMPS, N-vinylpyrrolidone (NVP), and SSS to control API filtration loss to about 10 mL at 240 °C. Ma et al. [44] prepared a bis-quaternary ammonium-type amphoteric copolymer (PADAN), which contains quaternary ammonium cations in its molecular structure. The polymer possessed enhanced adsorption on the surface of clay particles, inhibiting clay swelling and hydration by electrostatic and hydrogen bonding interactions even after aging at 200 °C. Liu et al. synthesized a non-toxic, high temperature polymer (BDF-100S) by combining AMPS, AM, Compound X, and Compound Y. BDF-100S effectively controlled the filtration loss at 200 °C. Additionally, organosilicon polymers can obtain higher temperature resistance due to Si-OH groups in the polymers. These groups allow the polymers to chemisorb onto clay particles, independent of temperature. Ban et al. [45,46] prepared an organosilicon filtration loss reduction agent (AATN) that can withstand high temperatures up to 180 °C. This agent effectively wraps around the bentonite and reduces the filtration loss of drilling fluids by forming a chemisorption layer.

Nanomaterials also have a wide use [47]. For instance, Liu et al. [48] used vinyltrimethoxysilane (A171) to modify the silicon nanoparticles through a free radical polymerization reaction with modified silicon nanoparticles, DMAM, and AMPS. The resulting product (NS-DA) demonstrates good filtration control and maintains stability at a high temperature of 180 °C. Xiong et al. [49] used N, N-dimethylacrylamide (DMAA), DMDAAC, SSS, AMPS, and PDDSA-MS, which was prepared from silicon dioxide (SiO_2_) modified with KH_570_. At 1 wt% of PDDSA-MS, the filtration loss significantly decreased to 8.8 mL after aging at 260 °C. PDDSA-MS composites have been successfully applied in water-based drilling fluids.

Looking for filtration loss control agents, which are easy to synthesize and have a high performance, has great significance for companies to reduce production costs and allow for better drilling operations. In this study, we prepared an ultra-high temperature resistant filter loss reduction agent via simple radical polymerization based on β-CD and KH_570_, SSS, and DMDAAC as raw materials. Cyclodextrins enable polymers to have good rheological properties, and organic compounds enhance the product’s solubility in water and stability at ultra-high temperatures. Then, we optimized the filtration and rheological properties of LY-2 in the drilling fluid by adjusting the proportions of different monomers and the crosslinker agent. The filtration properties of drilling fluid with LY-2 were assessed after aging at temperatures ranging from 200 °C to 260 °C. And we observed the system stability via the phenomenon of the LY-2 drilling fluid system after different times of resting. Finally, we compared LY-2 with other commercial filtrations in a regular water-based fluid system.

## 2. Results and Discussion

### 2.1. Optimization of Polymer Monomer Ratio

#### 2.1.1. Effect of Different Monomer Ratio on Particle Size

**Figure 1:**
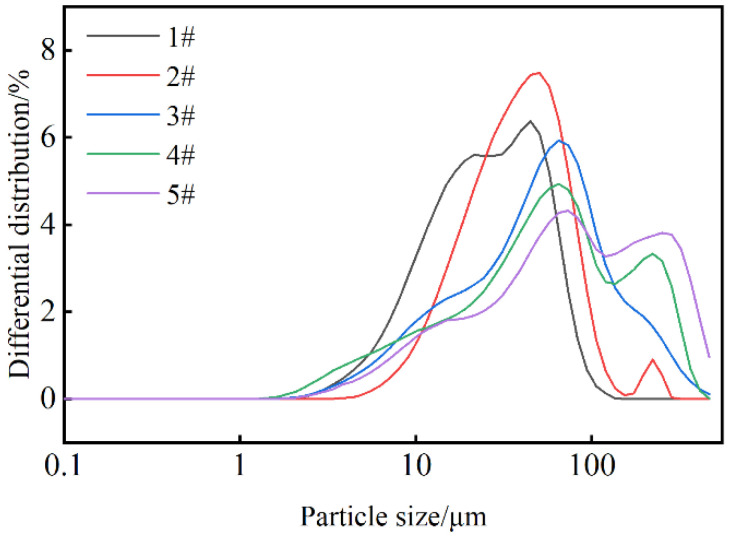
Different monomer ratios and their corresponding particle sizes at room temperature.

Figure 1 shows the particle size distribution of LY-2 polymers when the percentage of synthetic monomers is varied. The peak sizes of samples 1#, 2#, and 3# appeared at 47.4 μm, 51.6 μm, and 70.4 μm, respectively, and D50 sizes were 27 μm, 40.68 μm, and 50.4 μm. As the proportion of β-CD increases, the peak gradually shifts to the right, and the average size also increases. The peak sizes of samples 4# and 5# appeared at 68.65 μm and 75.41 μm, respectively, and D50 sizes were 59.75 μm and 81.67 μm, suggesting that the peaks shifted to the right and the average particle size increased as the proportion of EPI increased. Table 1 shows median particle size of LY-2 corresponding to different monomer proportions.

In addition, a double peak appeared at sizes larger than 100 μm while the height of the first main peak decreased, and with the increase in EPI content, the content of larger particles increased synchronously and gradually approached the height of the first main peak, indicating that the increase in the percentage of crosslinking agent has a greater impact on the volume distribution of the polymer. By controlling the dosage of β-CD and EPI, different particle sizes can be achieved, allowing for the regulation of product particle size. In practical applications, the selection of monomer ratios can be further optimized by considering the relationship between polymers D90, D75, D50, D25, and D10 and the pore size distribution in natural reservoirs [50]. This optimization is necessary to effectively block filtration loss channels, accelerate the formation of low-permeability filter cake, and quickly establish an initial filtration loss barrier on the wellbore wall.

#### 2.1.2. Influence of Different Monomer Ratios on BF Filtration Loss Properties

**Figure 2:**
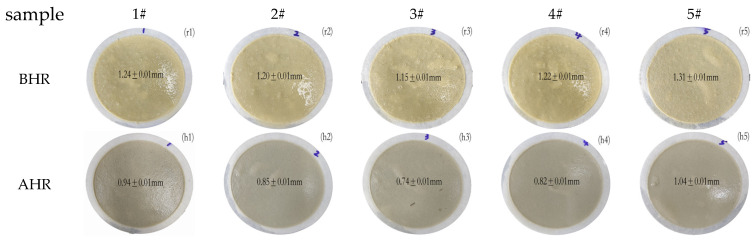
Different monomer proportions correspond to filter cake BHR and AHR at 200 °C.

From Figure 2, in the room temperature mud cake, it can be observed that some tiny particles can be seen on the bentonite filter cake; the product in the base drilling fluid is not completely dissolved. However, in AHR, the transparent particles completely disappear. At the same time, the thickness of the cake is significantly reduced compared to BHR, and it becomes denser and smoother. This phenomenon occurs because the high temperature damages the polymer’s micro-balloon structure, causing a decrease in particle size. It can increase the solubility of the polymers, which is more conducive to maintaining the rheological properties of the substrate, as well as producing a stronger adsorption capacity for bentonite. It also means the polymer has a better effect on AHR.

**Figure 3:**
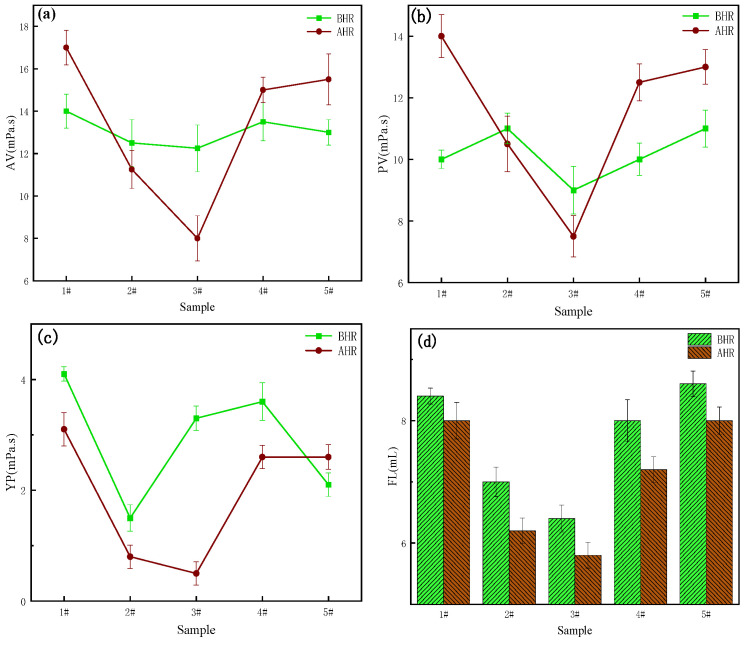
Effect of different monomer ratio products at 1% concentration on the properties of BF: (**a**) AV; (**b**) PV; (**c**) YP; (**d**) FL_API_.

In Figure 3, it is evident from 1#, 2#, and 3# that as the proportion of β-CD increases from 50% to 70%, the FL_API_ decreases from 8.4 mL to 6.4 mL BHR and from 8 mL to 5.8 mL AHR at 200 °C. At room temperature and AHR, the filtration loss gradually decreases with an increase in the cyclodextrin monomer ratio. This is because as the proportion of β-CD increases, the particle size distribution becomes multi-peak, allowing different polymer particle sizes to better fill in the gaps of bentonite. Additionally, the coarse particles can form a bridge structure, connecting with each other, while the fine particles fill in the gaps between the coarse particles, resulting in a low-permeability tight filter cake. This effectively seals the wellbore wall, reducing the filtration loss of the drilling fluid [51,52,53,54].

As observed in samples 1#, 4#, and 5#, increasing the proportion of crosslinker monomer EPI leads to larger particle size, resulting in a gradual increase in fluid loss. When the proportion of EPI increased from 15% to 25%, the amount of FL_API_ BHR increased to 8 mL and 9 mL, and AHR, the amount of FL_API_ increased from 7.2 mL to 8 mL at 200 °C. The filtration loss of the AHR becomes significantly lower than BHR due to the fact that the particle size of the polymer microspheres increases with the increase of crosslinker monomers and reduction in specific surface area, making it difficult to dissolve in the substrate and hinder adsorption. However, after aging, the three-dimensional microsphere structure of the product was destroyed, the polymer was transformed into a two-dimensional network structure, and carbon nanoparticles with different particle sizes were released. At the same time, a large number of hydroxyl functional groups are exposed, which increases the dissolution hydration and enables the polymer to better perform the function of reducing the amount of filtration loss.

Comparing the rheological data of five samples before and after aging, it can be found that the viscosity and rheological properties were maintained well after aging at 200 °C, and the rheological data of the system after aging at 200 °C were little changed compared with those of the samples before aging, which indicates that the filter loss reducer plays a better control role in stabilizing the rheological properties of drilling fluids. While the increase of β-CD monomers induced a slight decrease in the AV of the system and the increase of crosslinking monomers, the AV of the system increased slightly. This phenomenon was more obvious in the aged system. When the raw material monomer β-CD accounted for 70% of the system, the AV, PV, and YP were moderate, and the lowest amount of filtration loss was selected for subsequent testing. Finally, 3# is the best named LY-2 due to its moderate rheological performance and lowest filtration values.

### 2.2. Characterization of LY-2

#### 2.2.1. FTIR Analysis

**Figure 4:**
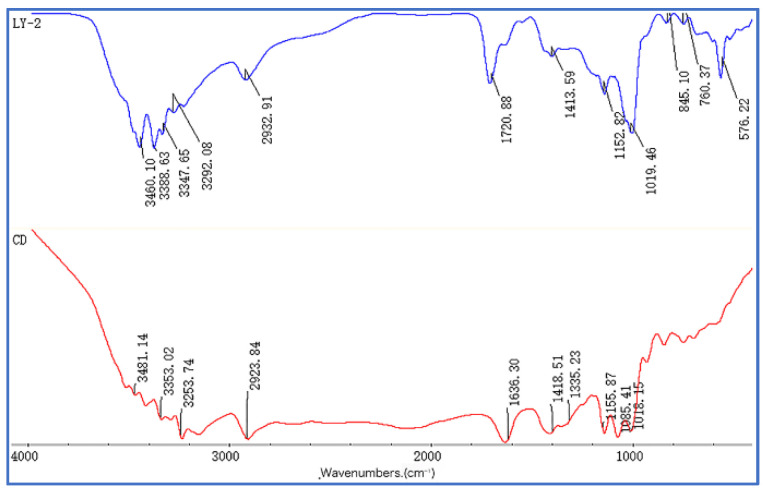
FTIR spectra of CD and LY-2.

The infrared spectrum of the CD and LY-2 polymer is shown in Figure 4. The peaks at 3202.8~3567.3 cm^−1^ and 1646.57 cm^−1^ correspond to the stretching and bending vibrations of the -OH bond in β-cyclodextrin [55]. The peak at 2932.91 cm^−1^ represents the stretching vibration of the sp^3^ hybridized carbon-hydrogen bond. In LY-2, the infrared spectrum also contains the corresponding peak. Additionally, the peak at 1720.88 cm^−1^ corresponds to the stretching vibration of the carbonyl-C=O bond [56]. The peak at 1413.59 cm^−1^ shows the -O-H bond in the carboxyl group, while the peak at 1343.24 cm^−1^ represents the absorption of the -C-N bond. The vicinity of 1152.82 cm^−1^ exhibits the stretching vibration of the carboxyl group -C=O bond. The peaks at 1019.46 cm^−1^ and 682.34 cm^−1^ indicate the presence of the -S=O and -C-S bonds in sulfonate, respectively [57]. Moreover, the peak at 845.10 cm^−1^ represents the Si-O bond in KH_570_ [58]. And 760.37 cm^−1^ corresponds to the in-plane bending vibration of the benzene ring. The peak at 576.22 cm^−1^ indicates the -C-O bond in the hydroxyl group. Lastly, the characteristic absorption peak at 501.08 cm^−1^ confirms the presence of the C-Cl bond, which demonstrates the successful monomer reaction and the production of the target product.

#### 2.2.2. Thermal Stability Analysis

**Figure 5:**
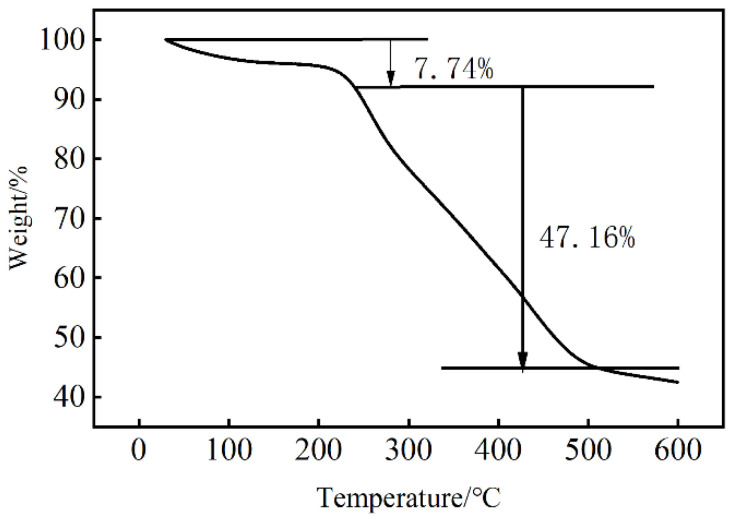
TGA curve of LY-2.

Figure 5 illustrates the thermal weight loss curve of LY-2. In the initial stage, there is a 7.74% weight loss from room temperature to 238 °C. It is attributed to the volatilization of crystalline water that was adsorbed by the polymer. It indicates that the polymer remains stable during this stage, with no rupture of functional groups or crosslinking structure. Starting at 238 °C, the weight loss of the filter loss reducer becomes consistent as the molecular structure starts to decompose, and the cyclodextrin crosslinking section of the chemical bond undergoes breakage, leading to the first signs of damage. Between 238 °C and 511 °C, there is a 47.16% mass loss. The weight tends to stabilize in the temperature range of 511 °C to 600 °C. Heat analysis data reveal that the complete decomposition temperature of the polymer is more than 600 °C, highlighting the excellent temperature resistance of LY-2.

### 2.3. Performance Evaluation of the LY-2

#### 2.3.1. Zeta Potential

**Figure 6:**
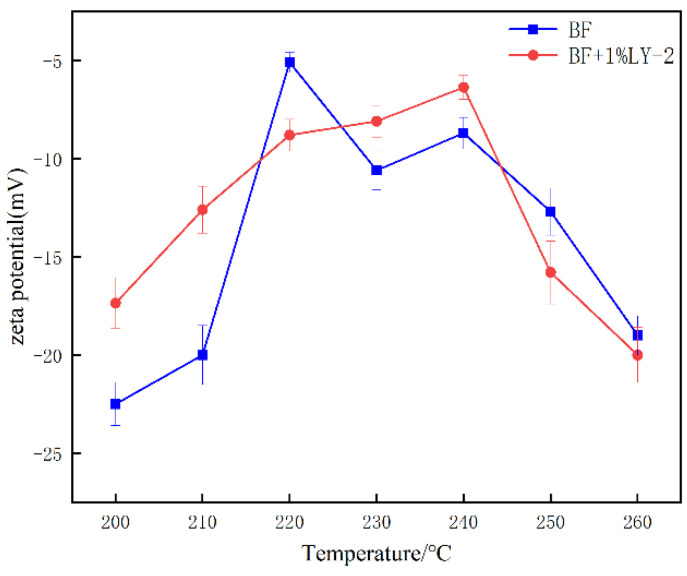
zeta potential of BF and BF+1%LY-2.

**Figure 7:**
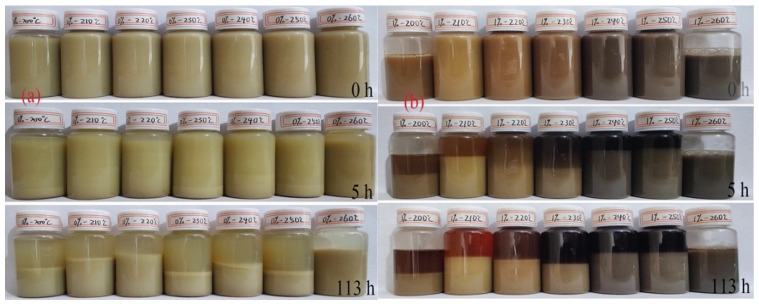
Stratification of BF and BF+1% LY-2 system: (**a**) BF; (**b**) BF+1% LY-2 system.

Figure 6 illustrates the changes in the Zeta potential of BDF and BDF+1 wt% LY-2 system after hot rolling at different temperatures. The results indicate that the magnitude of the effect of LY-2 on the Zeta potential of the drilling fluids is not particularly significant. It could be due to the sulfonate ions and a large number of negatively charged hydroxyl functional groups on the polymer chain adsorbed on the bentonite, thus increasing the thickness of the hydration layer and preventing the aggregation of particles, and cationic monomers in the polymer will promote the aggregation of bentonite flakes, so the LY 2 impact drilling fluid’s Zeta potential is not particularly significant. For the same reason, comparing BF aged at different temperatures to BF+1 wt%LY-2 system reveals that the latter exhibits a more rapid increase in water volume within the first 5 h. This is supported by the data in Figure 7, which represents the percentage change in water content. Additionally, exceeding 240 °C, the zeta value of the added LY-2 tends to decrease compared with the BF system, enhancing the dispersion stability of the clay particles at ultrahigh temperatures and improving the ability to minimize losses.

#### 2.3.2. Sedimentation Stability Measurement

**Figure 8:**
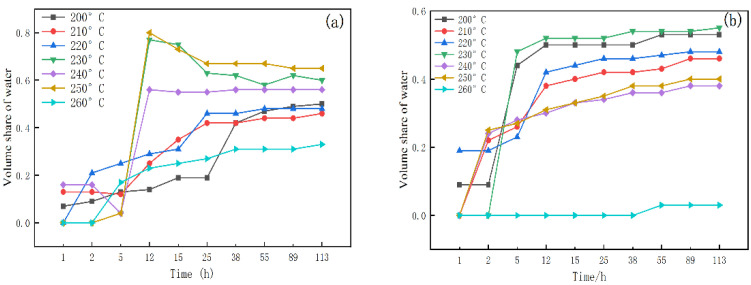
Volume share of water at BF and BF+1% LY-2 system: (**a**) BF; (**b**) BF+1% LY-2 system.

To assess the impact of polymer on the stability of the matrix, we conducted tests on sedimentation stability at various temperatures AHR. The bentonite matrix was aged alone, as well as with 1% LY-2, at temperatures ranging from 200 °C to 260 °C for 16 h. Subsequently, a portion of the aged product system was placed in a transparent vial and observed over different time intervals for delamination. The findings are displayed in Figure 7a,b. The volume of the water layer that separated after each time interval was compared to the volume of the sampled system, as shown in (Figure 8a,b).

It can be seen in Figure 7a,b that the color of the aging system gradually deepens as the aging temperature increases. This is because the cyclodextrin crosslinker produces carbon nanoparticles. The change in color of the solution also makes it easier to see the degree of polymer decomposition. Especially at temperatures up to 260 °C, the system does not delaminate even after more than 113 h of standing time. This is likely because 1% of the added LY-2 can completely decompose and release carbon nanoparticles at this temperature. The adsorption of bentonite by hydrated groups and a large number of hydroxyl functional groups further disperses the clay particles, which makes it difficult for them to gather and form agglomerates, thus increasing the stability of the system and ensuring good filtration performance. In contrast, at 260 °C, the BF (AHR) shows a clear water layer, which indicates that the water molecules under the ultra-high temperature movement of the water molecules completely separated from the bentonite. Because the BF at high temperatures is a very complex process containing high temperature desorption, high temperature aggregation, as well as high temperature passivation, it follows different temperatures after aging the BF after 100 h of resting, turbidity, and layering produced differences and are not very regular. And from Figure 7, we can see the addition of 1% polymer for the BF has a better role in maintaining the stability of the system.

Figure 8a,b shows that the BF has a slower delamination rate compared to the system with 1% LY-2 added. After 5 h of resting, the water fraction of the BF after aging at temperatures from 200 °C to 260 °C is as follows: 13%, 12%, 25%, 4%, 4%, 4%, and 17%. In comparison, the water fraction of the 1% LY-2 system is 44%, 26%, 23%, 48%, 28%, 27%, and 0% of the total volume of the system. When the resting time exceeds 72 h, the degree of delamination of the system begins to stabilize. At this point, the water volume for the BF after hot rolling at different temperatures is 49%, 44%, 48%, 62%, 56%, 65%, and 31%, and 53%, 46%, 48%, 54%, 38%, 40%, and 3% for the 1% LY-2 system. This indicates that the presence of the polymer accelerates the delamination of the system. However, after a longer period, the degree of delamination will be less than the BF. Therefore, LY-2 enhances the stability of the system to a certain degree, especially at higher temperatures.

#### 2.3.3. Rheological and Loss Control Properties

**Figure 9:**
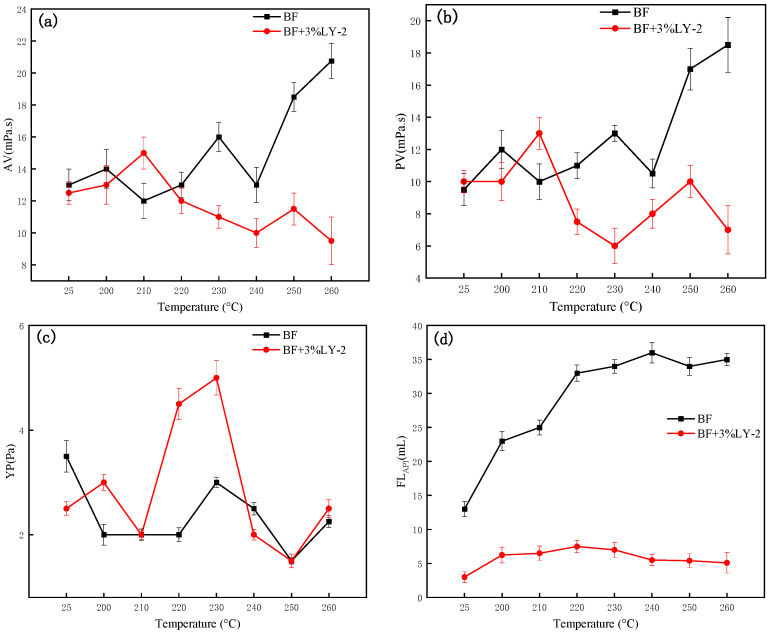
Effect of temperature on the rheological and loss control properties of BF and 3% LY-2 added to BF system: (**a**) AV; (**b**) PV; (**c**) YP; (**d**) FL_API_.

The measurements were taken for both the BF and the BF with 3% LY-2, before and after aging at different temperatures: 200 °C, 210 °C, 220 °C, 230 °C, 240 °C, 250 °C, and 260 °C. The readings of Φ300 and Φ600 were recorded, then AV, PV, and YP were calculated. From the experimental results in Figure 9, the AV and PV of the BF initially decreased, then increased significantly when the temperature exceeded 240 °C. This can be attributed to the increase in the dehydration of clay particles at ultra-high temperature environments and the decrease in specific surface area of the clay particles, which promoted the end-to-face and end-to-end bonding of clay particles, forming a “card-type” bond. In addition, the over-dispersed clay particles will gather and absorb generous water, decrease the free water, and indirectly lead the solids to increase in high temperature, high density, water-based drilling fluids, exhibiting high temperature thickening characteristics.

The addition of LY-2 actually reduces the viscosity of the BF acting as a viscosity reducer compared to the base drilling fluid after aging at 25 °C, 200 °C, 220 °C, 230 °C, 240 °C, 250 °C and 260 °C. The main reason can be attributed to three points. Firstly, the monomer KH_570_ in LY-2 has a silicone-oxygen bond, and the bentonite contains silicon hydroxyl to form a more stable silicone ether bond. It can increase the polymer’s adsorption capacity on the bentonite through chemical adsorption, thus inhibiting the bentonite’s ability to hydrate, thickening the clay hydration film, and increasing hydration membrane repulsive force, thus preventing clay particles agglomeration under high temperature and pressure. Secondly, the cationic monomers contained in LY-2 were able to adsorb on the clay end face, preventing the formation of a bentonite reticulation structure, which can prevent bentonite aggregation [37,43]. Thirdly, when the bentonite reticulation structure is formed at high temperatures, the carbon nanoparticles fully released by the polymer can cause the reticulation structure to be disassembled in the flow, restoring the bentonite to a granular or lamellar structure, thus keeping the rheological properties stable. Overall, the polymer can maintain the adsorption of bentonite at high temperatures through the interactions of chemical bonding, hydrogen bonding, and electrostatic interactions at high temperatures, effectively slowing down the desorption effect at high temperatures, and at the same time, it has a certain stabilizing and regulating effect on the rheological properties of drilling fluids through the carbon nanoparticles of varying sizes.

Compared to the addition of 1 wt% LY-2, increasing the LY-2 addition to 3% only slightly increased the viscosity of the BF. This indicates that the polymer has a good effect on retaining the viscosity of drilling fluid. Furthermore, when 3% LY-2 was added to the BF during the dissolution operation, it dissolved in less than three minutes, which indicated that LY-2 could easily dissolve and had little effect on drilling fluid rheological properties.

In comparison to the BF, the YP of the BF with 3 wt% LY-2 polymer added slightly increased after aging at different temperatures. As the temperature increased to 260 °C, the filtration loss of the BF with 3 wt% LY-2 remained around 5 mL, with only a slight increase, indicating that the loss-reduction remained a good performance at high temperatures. The BF with 3 wt% LY-2 system had a filtration loss of 3 mL, which was 77% lower than that of the blank system sample (13 mL) at ambient temperature, indicating that LY-2 has a better filtration loss reduction effect on the BF at room temperature. After aging at 260 °C for 16 h, the filtration loss of the same sample was 5.1 mL, which was 85.4% lower than the BF aged at the same temperature (35 mL). These test results demonstrate that LY-2 has an excellent filtration loss reduction ability against ultra-high temperatures.

#### 2.3.4. Physical and Microcosmic Images of Filter Cake

**Figure 10:**
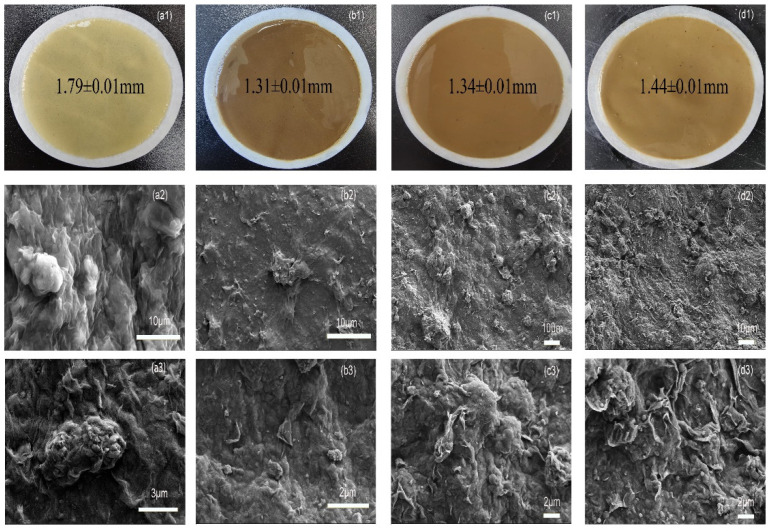
Physical and SEM images of API loss filter cake before and after addition of 3%LY-2 hot roll to the base fluid: (**a1**–**a3**) BHR; (**b1**–**b3**) 200 °C; (**c1**–**c3**) 230 °C; (**d1**–**d3**) 260 °C.

To investigate the influence of polymers on bentonite after undergoing high temperature thermal rolling, the filter cake was examined using SEM. Figure 10(a1–a3) shows the microcosmic image of the 3% LY-2 added the BF filter cake at room temperature. The polymer and bentonite have fused completely, with the bentonite crystalline layer being fully covered and wrapped, resulting in a crack-free surface. By magnifying the SEM image, one can observe the presence of large particles forming a crosslinked spherical network structure. These spheres are closely connected to the bentonite surface structure, surrounded by extending branches that penetrate into the interspaces of bentonite. This process effectively seals the cracks in the filter cake and is highly beneficial in ensuring the excellent performance of the filter cake.

When subjected to temperatures exceeding 200 °C during thermal tumbling, the filter cake exhibited a brown color (Figure 10(b1,c1,d1)), which resulted from the decomposition and carbonization of the polymer at high temperatures. Figure 10(b1–b3) illustrates the filter cake after aging at 200 °C; compared to the filter cake aged at room temperature and higher temperatures, the surface layer of the filter cake is flatter and more delicate. The polymer-coated bentonite surface contracts and the reticulation structure integrates almost seamlessly with the bentonite layer. There are only a few dispersed small particles on the surface of the filter cake, and the veins are not very prominent. As the aging temperature increases, the mesh veins become more distinct, and the structure remains intact. This enables the polymer to connect the bentonite platelets through ionic bonding and hydrogen bonding, which helps to control the filtration loss of the drilling fluid. In the SEM image of the filter cake subjected to rolling at 230 °C (Figure 10(c1–c3)), a large number of spherical particles can be observed dispersed on the surface of the filter cake, with a clearer reticulation structure compared to the cake aged at 200 °C. These particles adhere tightly to the surface of the bentonite. The filter cake surface is covered with polymers. With further magnification of the SEM image, it becomes apparent that there are numerous spheres of different sizes, as well as some broken small particles. Meanwhile, these nanoparticles have a sealing effect on the voids of the filter cake, promoting the formation of a low-permeability dense filter cake, improving cake quality, increasing cake compressibility, and thus reducing filtration loss in high temperature environments.

In Figure 10(d1–d3), the SEM image of the filter cake after hot rolling at 260 °C reveals a denser distribution of particles of different sizes, along with the clear appearance of reticular vein drops. Increasing the magnification, it is more evident that a significant number of particles with varying sizes have emerged, which are hydrothermal carbonization products of cyclodextrins. It can maintain good stability and mechanical strength under high temperature environments, also effectively seal in the interstitial space of bentonite clay during filtration, and block the reduction in filtration loss. Additionally, the large side-chain rigidity groups contained in the SSS and silicone monomers further enhance the temperature resistance of the polymers, ensuring that the polymers maintain their loss reducing properties at ultra-high temperatures. Still, at this time, no flaky or rod-like clay mineral structure appeared on the surface of the filter cake, and there were no obvious fracture surfaces, indicating that LY-2 can effectively control filtration loss even after hot rolling at 260 °C. The microcosmic images of the filter cake demonstrate that the polymer particles, with a complex chain structure, are distributed in the voids and on the surface of the filter cake. The carbon nanoparticles, resulting from the decomposition of cyclodextrin crosslinked microspheres, promote the formation of a smooth, homogeneous, and dense filter cake by bridging and blocking the pore space. Additionally, they prevent the agglomeration of bentonite at high temperatures, which would otherwise lead to the formation of large pore spaces and cracks. As a result, the passage of free water in the drilling fluid is further reduced, thus maintaining the drilling fluid and improving its filtration loss performance.

#### 2.3.5. Comparing LY-2 with Other Anti-Temperature Filtrate Reducers

**Figure 11:**
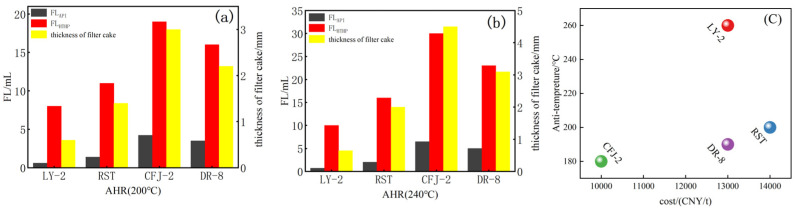
Comparison of different Filter Loss Reducers: (**a**) AHR at 200 °C; (**b**) AHR at 240 °C; (**c**) Cost comparisons.

We note that 1 wt% of various anti-temperature filter loss reduction agents were added to the SWBF system. After aging at 200 °C for 16 h, the FL_HTHP_, FL_API_, and thickness of filter cake were measured. The experiment results are shown in Figure 11a. It can be observed that CFJ-2-containing drilling fluid had the highest FL_API_ at 3.2 mL and FL_HTHP_ at 15.8 mL. RST had better anti-temperature filtration loss reduction ability compared to CFJ-2 and DR-8. However, among the four filtration reducer agents used, LY-2 exhibited the best anti-temperature filtration loss reduction performance with API filtration loss of 0.6 mL and low FL_HTHP_ of 7 mL. Additionally, the filter cake was thin and dense, with a thickness of only 0.6 mm. When the temperature gets 240 °C shown in Figure 11b, CFJ-2, DR-8, and RST produced a certain increase in the filtration loss volume, and FL_API_ for RST increased from 3.2 to 6.5mL, FL_HTHP_ increased from 15.8 to 30 mL, which has a smaller volume compared with other two filter loss reduction agents, and it means RST has a good resistance to temperature. While FL_API_, FL_HTHP_, and the cake thickness of LY-2 remained stable and almost unchanged, suggesting that LY-2 has superior filtration loss control ability in the SWBF. In Figure 11c, we can see that LY-2 has the highest temperature resistance ability with a relatively lower cost compared to RST.

## 3. Materials and Methods

### 3.1. Materials and Instruments

Sodium hydroxide (NaOH, 98%), β-cyclodextrin (β-CD, 98%), epichlorohydrin (EPI, 98%), anhydrous ethanol (EtOH, 99.5%), acetone (AC, 99.5%), sodium bisulfite (NaHSO_3_, 98%), sodium p-styrenesulfonate (SSS, 98%), ammonium persulfate (APS, 98%), 3-(Trimethoxymethylsilyl)propyl methacrylate (KH_570_, 97%), products of Shanghai McLean Biochemistry and Technology Company (Shanghai, China). Dimethyldiallyl ammonium chloride (DMDAAC, 60%), product of Shanghai Bide Pharmaceutical Technology Company (Shanghai, China). Anti-temperature, anti-salt, anti-collapse filtration loss reducing agent DR-8 was supplied by Binzhou Derun Chemical Company (Binzhou, China). Anti-temperature filter loss reducing agent CFJ-2 was provided by Langfang Qingxing Chemical Company (Langfang, China). Anti-temperature and anti-salt filter loss reducing agent RST was provided by Chengdu Dedao Industrial Company (Chengdu, China).

### 3.2. Synthesis of Copolymer LY-2

We note that 5% SSS, 5% DMDAAC, 30% KH_570_, and 50% β-CD were sequentially added to distilled water in a three-necked flask connected to an electric stirrer, and 40% NaOH aqueous solution was used to adjust the solution pH to 8–10. Afterward, 10% EPI was added dropwise at 35 °C for 1.5 h at N_2_ atmosphere. Then, APS and NaHSO_3_ (3:1) were added to the system, and the reaction temperature was raised to 60 °C for 4 h. To obtain pure solids, the low-viscosity gel product was washed several times with ethanol/acetone (1:1 *v*/*v*), dried at 65 °C for 12 h, and then pulverized to obtain an LY-2 filtration loss agent.

### 3.3. Characterization of LY-2

The particle size distribution of the polymers was tested using the BT-802 laser particle size analyzer produced by Dandong Baxter Instrument Company (Dandong, China). Add 1% LY-2 to 30 mL of distilled water and then ultrasonicate for 0.5 h to achieve uniform dispersibility at room temperature.

The LY-2 samples were pressed into tablets with potassium bromide (KBr), and the functional group characterization was carried out using a Nicolet 710 infrared spectrometer manufactured by Thermo Fisher Scientific (Shanghai, China) at a scanning frequency of 500–4000 cm^−1^ and a scanning rate of 32 scans/min.

The thermal property of LY-2 was tested using the STA449C Thermogravimetric Analyzer from NETZSCH Scientific Instruments Company (Shanghai, China). Temperature ranges from 25 °C to 600 °C, with a heating rate of 10 °C/min, and the flow rate of nitrogen was 30 mL/min.

The microstructure of the surface of the filter cake was observed after lyophilization, slicing, and gold spraying in a vacuum environment with an ultra-high-resolution field emission scanning electron microscope, Apreo 2C, manufactured by Thermo Fisher Scientific (Shanghai, China).

The zeta potential was measured using a JS94K microelectrophoresis instrument manufactured by Shanghai Zhongchen Digital Technology Equipment Co., Ltd. (Shanghai, China) using a 1000-fold dilution of 1% LY-2 drilling fluid.

### 3.4. Drilling Fluid Formulation

To prepare the base fluid (BF), 4 wt% bentonite and 0.2 wt% sodium carbonate were dispersed in water, and the mixture was stirred at 4000 rpm for 2 h and allowed to hydrate for 24 h at room temperature [1]. To test the suitability of the prepared polymers in a field environment, a simplified water-based fluid (SWBF) system (Table 2) was prepared, with all components added sequentially and then hydrated for 24 h for the latter testing.

### 3.5. Evaluation of Rheological Properties

The rheological properties of the drilling fluid were evaluated using a six-speed rotational viscometer, model ZNN-D6A, manufactured by Qingdao Senxin Electromechanical Equipment Company. Viscosity data at 300 and 600 rpm (θ_300_, θ_600_) were recorded. Then, calculate the apparent viscosity (AV), plastic viscosity (PV), and yield point (YP) using Equations (1)–(3).

AV = θ_600_/2 (1)

PV = θ_600_ − θ_300_ (2)

YP = (θ_300_ − PV)/2 (3)

### 3.6. Evaluation of Filtration Reduction Performance

In accordance with the American Petroleum Institute (API) standards [59], FL_API_ was measured at 0.69 MPa pressure and room temperature using the ZNS-2 Medium Pressure Filtration Loss Flow Meter manufactured by Shandong Meike Instrument Company. FL_HTHP_ was measured after the drilling fluid system was aged, at 3.5 MPa and with the same temperature as the aging temperature using a GGS71-B high temperature and high-pressure loss through filtration meter manufactured by Qingdao Hengtaida Mechanical and Electrical Equipment Company (Qingdao, China). All measured data were collected three times to ensure the repeatability of the data, and the average value was taken.

## 4. Conclusions

In this work, we synthesized an ultra-high temperature resistant filter-loss reducer, LY-2, using KH_570_, DMDAAC, SSS, and β-CD as raw materials. FTIR revealed that the target product was obtained, and the TG curve showed excellent thermal stability.

The AV of the BF containing 3% LY-2 increased by about 2% compared to 1% LY-2 BHR, and AHR decreased by about 30%, thus revealing stable rheological properties. After aging at 260 °C/16h, FL_API_ was 5.1 mL, showing it can maintain good performance under 260 °C. The mechanism of LY-2 in controlling filtration loss was investigated by an SEM test, settling stability measurements and zeta potential analysis. The results showed that LY-2 accelerated the rate of delamination of the BF, reduced the degree of delamination, and had little effect on the zeta potential of the BF. At high temperatures, carbon nanoparticles released from polymer decomposition can adsorb and seal cracks in the mud cake to form a dense filter cake and reduce filtration loss. Additionally, cost comparisons showed that LY-2 breaks the upper limit of ordinary commercial filter loss reducer’s temperature resistance with a lower cost.

LY-2 has a great advantage in its easy preparation process. And it does not produce any irritating odor during use. Notably, the chosen ratio of cyclodextrin to epichlorohydrin is relatively ideal, and the monomer ratio can be further optimized for different practical environments.

## Figures and Tables

**Table 1:** Median particle size of LY-2 corresponding to different monomer proportions.

Sample	1#	2#	3#	4#	5#
ratio	β-CD50 wt% EPI 10 wt%	β-CD60 wt% EPI 10 wt%	β-CD 70 wt% EPI 10 wt%	β-CD 70 wt% EPI 15 wt%	β-CD 70 wt% EPI 25 wt%
D50/μm	27	40.68	50.4	59.75	81.67

**Table 2:** Simplified water-based fluid (SWBF) formulation.

Additive	Concentration (wt%)
bentonite	4
Na_2_CO_3_	0.2
NaOH	0.5
sulfonated asphalt	2~3
polymerized alcohols	0.3~0.5
sulfonated lignite resins	1~3
high temperature stabilizers	0.1
film-forming sealer	1~2
lubricants	2
shale inhibitors	1~3
potassium chloride	7

## Data Availability

The original contributions presented in the study are included in the article, further inquiries can be directed to the corresponding author.
